# The Ectopic Expression of the *MpDIR1(t)* Gene Enhances the Response of Plants from *Arabidopsis thaliana* to Biotic Stress by Regulating the Defense Genes and Antioxidant Flavonoids

**DOI:** 10.3390/plants13192692

**Published:** 2024-09-25

**Authors:** Mingzheng Duan, Liuyuan Bao, Momina Eman, Duo Han, Yongzhi Zhang, Bingsong Zheng, Shunqiang Yang, Muhammad Junaid Rao

**Affiliations:** 1 College of Agronomy and Life Sciences, Zhaotong University, Zhaotong 657000, China; duanmingzheng@ztu.edu.cn (M.D.); 47015@ztu.edu.cn (L.B.); han860401@ztu.edu.cn (D.H.); 34006@ztu.edu.cn (Y.Z.); 2 State Key Laboratory of Subtropical Silviculture, College of Forestry and Biotechnology, Zhejiang A & F University, Hangzhou 311300, China; emomina007@gmail.com (M.E.); bszheng@zafu.edu.cn (B.Z.); 3 Institute of Pure & Applied Biology (IP&AB), Bahauddin Zakariya University, Multan 60800, Punjab, Pakistan; 4 State Key Laboratory for Conservation and Utilization of Subtropical Agro-Bioresources, College of Agriculture, Guangxi University, 100 Daxue Rd., Nanning 530004, China; 5 Key Laboratory of Horticultural Plant Biology (Ministry of Education), Key Laboratory of Biology and Genetic Improvement of Horticultural Crops (Ministry of Agriculture), Huazhong Agricultural University, Wuhan 430070, China

**Keywords:** citrus, *Arabidopsis*, biotic stress, gene expression, stress response mechanisms, salicylic acid

## Abstract

The Defective in Induced Resistance 1 (*DIR1*) gene, a member of the lipid transferase proteins (LTPs), plays a crucial role in plant defense against pathogens. While previous transcriptomic studies have highlighted the significant expression of citrus LTPs during biotic stress, functional annotations of LTPs in the *Citrus* genera remain limited. In this study, we cloned the *Murraya paniculata DIR1* (*MpDIR1(t))* gene and overexpressed it in *Arabidopsis thaliana* to evaluate its stress response mechanisms against biotic stress. The transgenic *Arabidopsis* lines showed fewer disease symptoms in response to *Pseudomonas syringae* (*Pst* DC3000) compared to wild-type *Arabidopsis*. Defense and pathogenesis-responsive genes such as *PR1*, *PR4*, *PR5*, and *WRKY12* were significantly induced, showing a 2- to 12-fold increase in all transgenic lines compared to the wild type. In addition, the *Pst* DC3000-infected transgenic *Arabidopsis* lines demonstrated elevated levels of flavonoids and salicylic acid (SA), along with higher expression of SA-related genes, compared to the wild type. Moreover, all transgenic lines possessed lower reactive oxygen species levels and higher activity of antioxidant defense enzymes such as superoxide dismutase, peroxidase, and catalase under *Pst* DC3000 stress compared to the wild type. The up-regulation of defense genes, activation of the SA pathway, accumulation of flavonoids, and reinforcement of antioxidant defense mechanisms in transgenic *Arabidopsis* lines in response to *Pst* DC3000 underscore the critical role of *MpDIR1(t)* in fortifying plant immunity. Thus, *MpDIR1(t)* constitutes a promising candidate gene for improving bacterial disease resistance in commercial citrus cultivars.

## 1. Introduction

Plants possess a defense mechanism known as systemic acquired resistance (SAR), which protects them from a wide range of pathogens via signal transduction from infected to non-infected parts of the plant following a primary inoculation [1]. A key protein involved in SAR signal transduction is Defective in Induced Resistance 1 (*DIR1*) protein, which is expressed in all living cells [2]. *DIR1* is a lipid transfer protein that plays a crucial role in plant defense signaling by facilitating the movement of lipid-based molecules between different parts of the plant, essential for systemic acquired resistance [2]. As a lipid transfer protein (LTP), *DIR1* aids in the long-distance signaling of defense-related molecules such as azelaic acid (AzA), dehdryoabietinal (DA), glycerol-3-phosphate (G3P), and methyl salicylate (MeSA). These molecules are vital in amplifying the defense signals in distant tissues, ensuring that the entire plant can respond to pathogenic attacks [3]. The coordinated action of these signals enables the plant to effectively combat and resist subsequent pathogen attacks, thereby enhancing its overall resilience and health. Mutants deficient in *DIR1* exhibit a SAR-deficient phenotype [4]. Homologs of *DIR1* have been identified in *Arabidopsis thaliana* [2,3] and appear to be conserved in plant species such as tobacco (*Nicotiana tabacum*) [5] and cucumber [6]. Functional analyses have revealed that orthologs of *DIR1* also exist in tomato and soybean [6]. Studies indicate that the *DIR1* gene is conserved across various plant species, including citrus, and is expressed in response to pathogen infection and other stress conditions, underscoring its role in the citrus plant’s defense mechanisms [7]. The *DIR1* protein, belonging to the LTP family, produces a strong mobile signal that is activated locally after local infection and subsequently moves through the phloem to induce SAR in distant uninfected *Arabidopsis* leaves [4,8]. Furthermore, the resistance-promoting activity of molecules such as AzA, DA, G3P, and MeSA all require a functional DIR1 protein [2].

Plant species possess different sets of genes that respond to a variety of abiotic and biotic stresses, providing local or systemic defense mechanisms [9,10]. These defense genes usually belong to the WRKY transcriptional factors, PR (pathogenesis-related) proteins, and PI (protease inhibitors) gene families and are also associated with secondary metabolites that produce antimicrobial compounds [11,12,13,14]. The WRKY, PR, and PI transcription factor families, along with the *PAL* (phenylalanine ammonia-lyase) gene, play crucial roles in plant biotic stress defense mechanisms. The expression of these defense genes is highly dependent on the type of pathogen and varies among species [15]. Notably, several transcriptomic studies on citrus greening disease (CGD) have revealed that citrus species stimulate a number of genes belonging to the WRKY, PR, and secondary metabolic pathways, such as *PAL* gene categories, in response to CGD [16,17,18]. The *PAL* gene encodes an enzyme that catalyzes the conversion of phenylalanine to cinnamic acid, a key step in the phenylpropanoid pathway. This pathway leads to the production of a diverse group of secondary metabolites, including salicylic acid and various antioxidant flavonoids [19,20]. Generally, citrus species resistant to the CGD pathogen *Candidatus* Liberibacter asiaticus (*C*Las) exhibit a rapid and resilient response to CLas invasion (prior to *C*Las stability), whereas *C*Las-susceptible species show a weak or delayed activation of defense gene [18,21]. Meanwhile, *C*Las induces certain virulence proteins and enzymes that degrade salicylic acid and its derivatives, contributing to the failure of the host defense system [22,23] and resulting in serve symptoms [24].

As no CGD-resistant germplasm has been identified within the *Citrus* genus, some promising citrus relatives have shown resistance to CGD disease. Notable examples include orange jasmine (*Murraya paniculata*) [25] and *Poncirus trifoliata* [26], as well as primitive citrus such as papeda (*Citrus ichangensis*) [21] and the semi-tolerant *Atalantia buxifolia*, which exhibits mild symptoms [27,28]. Conversely, pummelo (*Citrus maxima*) [29] is susceptible, while sweet orange (*Citrus sinensis*) and mandarins (*Citrus reticulata*) are considered highly susceptible citrus species [28]. CGD-resistant and susceptible species exhibit the activation of different set of genes, metabolites, and defense pathways in response to *C*Las progression [21]. The high expression of certain genes in resistant species, as opposed to their lower expression in susceptible citrus species, highlights promising candidate genes potentially involved in resistance against *C*Las. The primary objective of this study was to determine whether the overexpression of the *MpDIR1(t)* gene could confer enhanced biotic stress resistance in transgenic *Arabidopsis thaliana* by modulating the expression of defense-related genes and the biosynthesis of antioxidant flavonoids. This study involved the assessment of gene expression profiles, salicylic acid determination in transgenic lines, antioxidant flavonoids, antioxidant enzymatic defense assays, and biochemical attributes of transgenic lines to quantify the levels of defense-related metabolites. Through this research, we aimed to better understand the mechanisms underlying biotic stress resistance and potentially develop crops with improved resistance to bacterial diseases in commercial citrus cultivars.

## 2. Results and Discussion

### 2.1. Gene Expression and Sequence Variation of DIR1 Gene between CGD-Resistant and Susceptible Species

**Figure 1:**
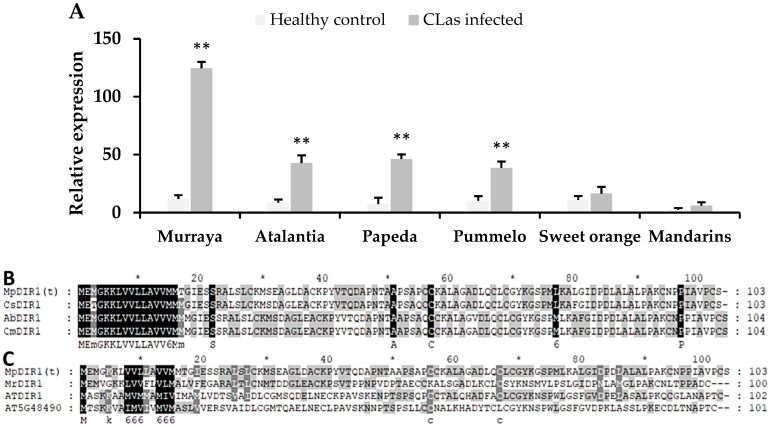
Gene expression and amino acid sequence analysis of *DIR1* gene. (**A**) Our gene expression pattern of the *DIR1* gene in six different citrus species (with and without *C*Las inoculation); (**B**) an amino acid sequence analysis of the *DIR1* homologous genes cloned from *Citrus reticulata* (*CsDIR1*), *Atalantia buxifolia* (*AbDIR1*), and *Citrus maxima* (*CmDIR1*); (**C**) the *MpDIR1(t)* amino acid sequence compared with its homologous genes from other plants (MR: *Morella rubra*, AT: *Arabidopsis thaliana*) Five-pointed star * in (**B**,**C**) marks every 10th amino acid for sequence counting. Healthy control: without *C*Las; *C*Las-infected: 4 weeks post inoculation with *C*Las bacteria. Each value is the mean of three biological replicates. A Student’s *t*-test was used to compare the gene expression of healthy and *C*Las-infected citrus at ** *p* < 0.01.

Wild citrus and its relatives exhibit varying degrees of resistance to the *C*Las pathogen. Previous transcriptomic studies have shown that citrus germplasm responds differently to *C*Las infection [7,28]. These studies revealed that the *DIR1* gene is significantly induced in *C*Las-resistant citrus species [30], whereas in susceptible species, such as sweet orange, the *DIR1* gene is either less expressed or down-regulated [30,31]. In our study, we used a resistant citrus relative, *Murraya paniculata*, and a highly susceptible species, *Citrus sinensis*, to examine the gene expression pattern of *DIR1* gene in response to *C*Las infection (Figure 1A). Upon *C*Las inoculation, the gene expression results indicated that the *DIR1* gene was significantly up-regulated in *Murraya paniculata* compared to sweet orange (Figure 1A).

Previous studies revealed that *C*Las-infected *Murraya paniculata* exhibits resistance or no symptoms and remains healthy in long-term field evaluation [25]. Additionally, *Murraya paniculata* has shown the lowest number of *C*Las bacterium titers compared to other citrus species [25,32]. These field and laboratory evaluations indicate that *Murraya paniculata* is resistant to CGD disease. The high expression of the *DIR1* gene following *C*Las invasion in *Murraya paniculata*, compared to sweet orange (Figure 1A), suggests a candidate gene that might confer resistance to bacterial stress. Moreover, we have cloned the *DIR1* gene to examine sequence variations among citrus species; the results revealed four amino acid variations between the resistant *Murraya paniculata MpDIR1(t)* gene and the susceptible *Citrus sinensis CsDIR1* gene (Figure 1B). Additionally, the *MpDIR1(t)* amino acid sequence showed the highest homology with *Morella rubra* and *Arabidopsis thaliana* (Figure 1C), which are involved in lipid transport, systemic acquired resistance, and salicylic acid-mediated defense signaling pathway activities [3].

### 2.2. Phylogenetic Analysis of MpDIR1(t) Gene

**Figure 2:**
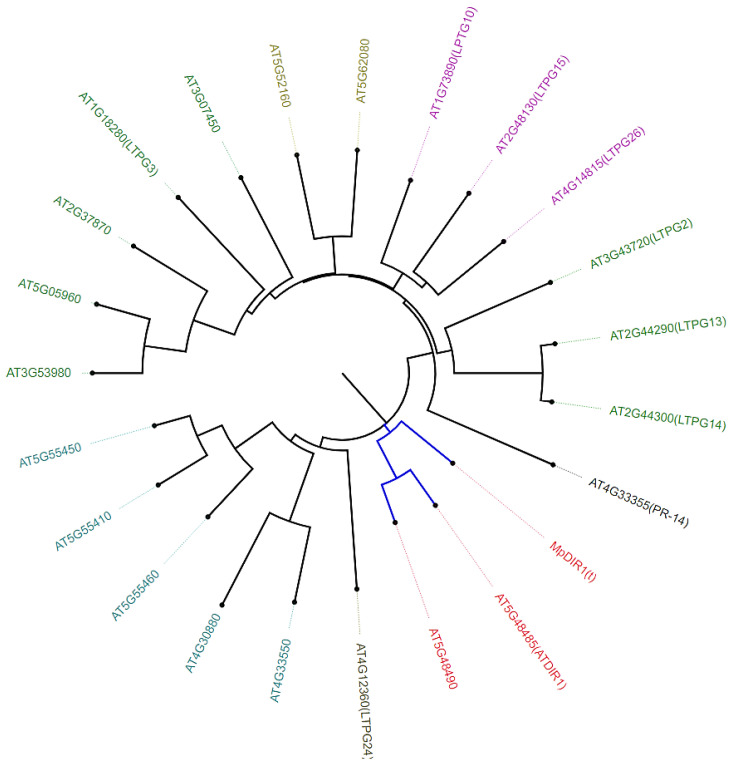
Phylogenetic analysis of the deduced protein sequence of the *MpDIR1(t)* gene with its homolog genes from *Arabidopsis thaliana*.

The *MpDIR1(t)* gene nucleotide sequence was subjected to a BLASTx (translated nucleotide to protein) analysis using an online tool to find homologous protein sequences in other plant species (https://blast.ncbi.nlm.nih.gov/Blast.cgi?PROGRAM=blastx&PAGE_TYPE=BlastSearch&LINK_LOC=blasthome (accessed on 20 January 2024)). In addition, the *MpDIR1(t)* gene was analyzed using the BLAST tool on the TAIR website (https://www.arabidopsis.org/ (accessed on 15 February 2024)). The phylogenetic analysis revealed that the *MpDIR1(t)* protein exhibits the highest similarity and homology to the *Arabidopsis DIR1* gene (Figure 2) [2,3,8,25,27]. Transient expression of the *Arabidopsis DIR1* gene has been shown to stimulate local or systematic resistance to biotic stress [8]. The phylogenetic and amino acid sequence analyses suggest that the *MpDIR1(t)* gene may function similarly to the *Arabidopsis DIR1* gene in systemic acquired resistance and the salicylic acid-mediated signaling pathways.

### 2.3. Ectopic Expression of MpDIR1(t) in Arabidopsis Confer Resistance to the Pst DC3000 Bacteria

**Figure 3:**
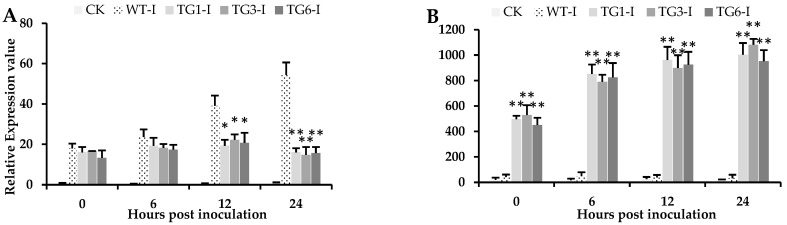
Inoculation of *Pst* DC3000 bacteria and gene expression analyses in the wild-type and transgenic lines. (**A**) Gene expression of the *Pst* DC3000 bacterial pathogen in *Arabidopsis* at different time points after inoculation, (**B**) Expression of the *MpDIR1(t)* gene in *Arabidopsis* at different time intervals after *Pst* DC3000 inoculation. CK: healthy control; WT-I: infected plants from the transgenic wild type (control); TG1-I: infected plants from transgenic line 1; TG3-I: infected plants from transgenic line 3; TG6-I: infected plants from transgenic line 6. Each value is the mean of three biological replicates. A Student’s *t*-test was used to compare transgenic *Arabidopsis* expressing *MpDIR1(t)*-TG and WT at * *p* < 0.05 and ** *p* < 0.01.

The *MpDIR1(t)* gene was significantly overexpressed in all transgenic *Arabidopsis thaliana* lines compared to the wild-type plants (Supplementary Materials; Figure S1). A *Pst* DC3000 bacterial suspension was infiltrated into the leaves of both wild-type and transgenic *Arabidopsis* expressing the *MpDIR1(t)* gene. Wild-type plants exhibited obvious symptoms of *Pst* DC3000 infection 12 h post-inoculation, with severe symptoms observed after 24 h. In contrast, all transgenic leaves displayed delayed symptoms between 24 to 48 h post-inoculation, with symptom intensity being slight in transgenic leaves. The gene expression analysis indicated that *Pst* DC3000 gene expression rapidly increased in the wild-type *Arabidopsis* leaves from 0 to 24 h post-inoculation with *Pst* DC3000 (Figure 3A). The increase in bacterial transcripts in both the transgenic and wild-type leaves was similar from 0 to 6 h post-inoculation, but from 6 to 24 h post-inoculation, the bacterial transcript levels were significantly lower in all transgenic lines compared to the wild type (Figure 3A). Conversely, all inoculated leaves of the transgenic lines showed a rapid increase in *MPDIR1(t)* expression from 0 to 24 h post-inoculation with *Pst* DC3000 (Figure 3B). These results suggest that the overexpression of the *MpDIR1(t)* gene plays a significant role in restricting the bacterial colonization of leaf tissues.

**Figure 4:**
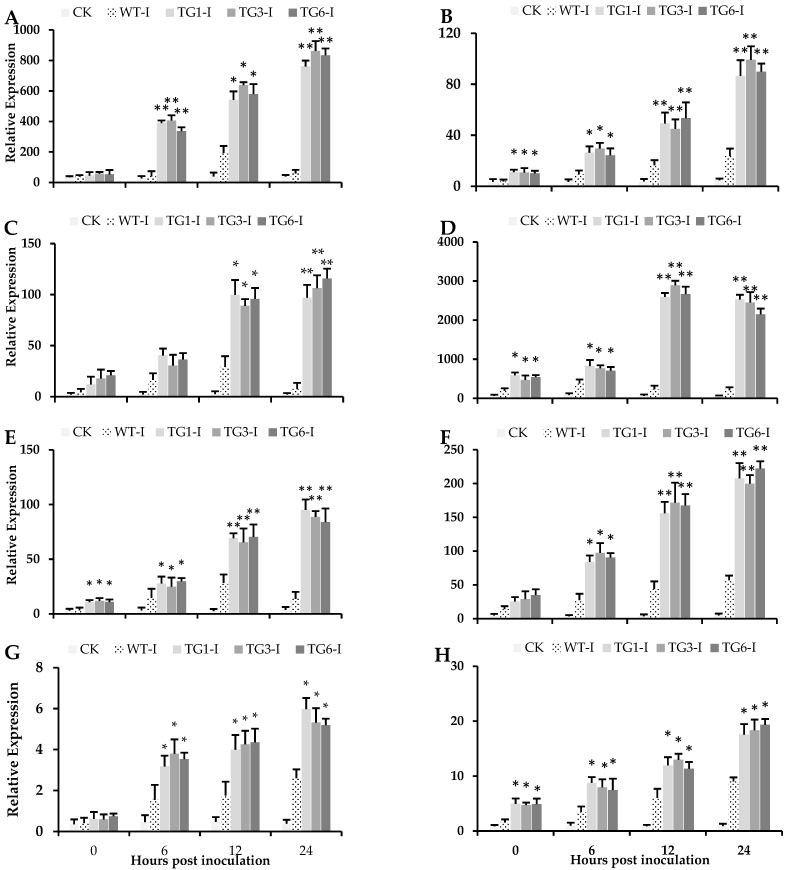
Expression pattern of pathogen-responsive and SA-mediated defense genes. (**A**) Protease inhibitors 1, *AtPI1*; (**B**) pathogenesis-related protein 1, *AtPR1*; (**C**) pathogenesis-related protein 2, *AtPR2*; (**D**) pathogenesis-related protein 4, *AtPR4*; (**E**) pathogenesis-related protein 5, *AtPR5*; (**F**) pathogenesis-related protein 10, *AtPR10*; (**G**) *AtWRKY12*; (**H**) phenylalanine ammonia lyase *AtPAL*. At: *Arabidopsis thaliana*; CK: healthy control; WT-I: infected plants from the wild type; TG1-I: infected plants from transgenic line 1; TG3-I: infected plants from transgenic line 3; TG6-I: infected plants from transgenic line 6. Each value is the mean of three biological replicates. A Student’s *t*-test was used to compare transgenic *Arabidopsis* expressing *MpDIR1(t)*-TG and WT at * *p* < 0.05 and ** *p* < 0.01.

The gene expression data indicated that the defense response genes, such as pathogenesis-related (PR) genes *AtPR1*, *AtPR4*, *AtPR5*, and *AtPAL*, were significantly induced in all transgenic *Arabidopsis* leaves (Figure 4). Previous studies have reported that a lipid transfer protein exhibits antifungal and antioxidant activity in transgenic wheat [33]. Pathogenesis-related genes including *PR1*, *PR2*, *PR4*, *PR5*, *PR10*, PI family genes, and WRKY DNA-binding transcription factor (*WRKY12*) genes, are involved in defense mechanisms and various processes in plants [34,35]. It has been previously documented that the *PAL* gene plays a role in the biosynthesis of salicylic acid in *Arabidopsis*, and a high expression of the *PR1* gene serves as a marker for the SA-mediated defense signaling pathway [36]. Furthermore, following inoculation with *Pst* DC3000, several genes associated with pathogenesis-related (PR) genes, protease inhibitors (PI) family, WRKY transcription factors, and *PAL* were significantly induced in the leaves of the transgenic lines compared to the wild type (Figure 4A–H). The WRKY, PR, and PI family genes are involved in both local and systematic defense responses to pathogen progression [11,12,13]. The expression of four additional genes, including *PI2* and *NPR1* (a positive regulator of SAR during biotic stress), is presented in the Supplementary Materials, Figure S2. Genes related to the PR, PI, and WRKY families are implicated in local or systemic defense against a variety of pathogens across many plant species [35,37,38]. The biosynthesis of salicylic acid and the induction of different defense responses (WRKY, PR, and PI) vary among plant species and are influenced by the lifestyle of the pathogen [15,39]. The intricate interplay between the PR, PI, and WRKY gene families underscores the complex defense mechanisms employed by plants to cope with pathogens. Species-specific variations in salicylic acid biosynthesis and defense gene induction highlight the adaptability of plants in responding to diverse bacterial disease challenges.

### 2.4. Higher Salicylic Acid, Antioxidants, and Flavonoid Compounds in Transgenic Lines Infected by Pst DC3000

**Figure 5:**
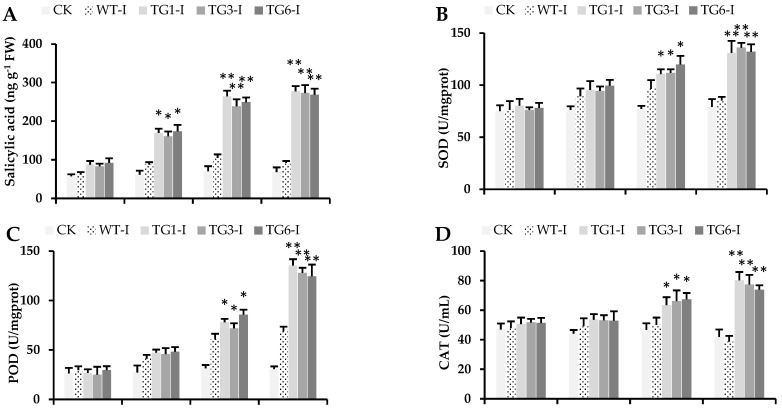

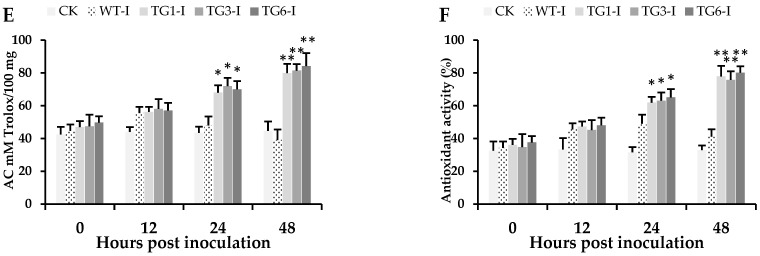
Salicylic acid and antioxidant enzymatic activities of *Arabidopsis* leaves injected with *Pst* DC3000. (**A**) Salicylic acid; (**B**) superoxide dismutase, SOD; (**C**) peroxidase, POD; (**D**) catalase, CAT; (**E**) antioxidant capacity (mM Trolox/100 mg); (**F**) antioxidant activity (%). CK: healthy control; WT-I: infected plants from the wild type; TG1-I: infected plants from transgenic line 1; TG3-I: infected plants from transgenic line 3; TG6-I: infected plants from transgenic line 6. Each value is the mean of three biological replicates. A Student’s *t*-test was used to compare transgenic *Arabidopsis* expressing *MpDIR1(t)*-TG and WT at * *p* < 0.05 and ** *p* < 0.01.

The concentration of SA was significantly higher in all transgenic leaves infected with *Pst* DC3000 6 h post-inoculation compared to the wild-type leaves (Figure 5A). Moreover, the *MpDIR1(t)* transgenic lines exhibited elevated levels of SA even in their healthy leaves relative to the wild-type leaves (Figure 5A). A high expression or overexpression of the *PAL* gene in *Arabidopsis* enhances SA accumulation, thereby increasing resistance to pathogen progression [19,40,41]. Notably, a significantly higher expression of the *PR1* gene was observed in all transgenic *Arabidopsis* lines compared to the wild type (Figure 4B). *PR1* is involved in the SA signaling pathway [36], and our results demonstrated elevated *PR1* gene expression in the transgenic lines (Figure 4B). The increase in SA levels (Figure 5A), coupled with the high expression of the *PAL* gene (Figure 4H) and the induction of several SA-related defense genes (Figure 4), clearly indicates that the *MpDIR1(t)* gene is involved in SA-mediated defense signaling. These findings elucidate the molecular mechanisms by which *MpDIR1(t)* enhances plant immunity and underscore its potential as a target for engineering SA-mediated defense strategies in crop plants.

**Figure 6:**
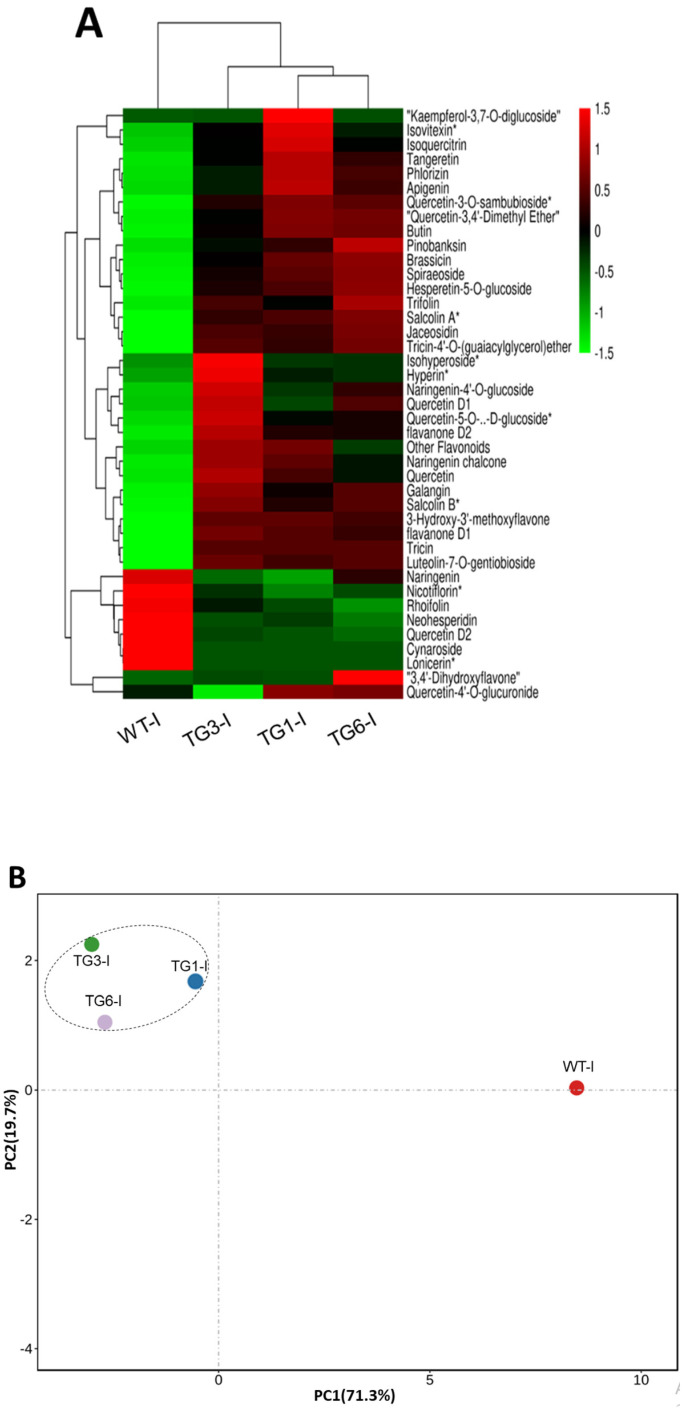
*Arabidopsis* leaf flavonoids infected with *Pst* DC3000 bacteria. (**A**) Hierarchical cluster analysis (HCA), where the columns signify Arabidopsis WT and transgenic lines and the rows represent flavonoid compounds (rows were normalized). (**B**) Principal component analysis (PCA). (*) means isomers of compound; WT-I: infected plants from the wild type; TG1-I: infected plants from transgenic line 1; TG3-I: infected plants from transgenic line 3; TG6-I: infected plants from transgenic line 6.

A high expression or overexpression of the *PAL* gene in *Arabidopsis* enhances flavonoids biosynthesis and leads to the accumulation of antioxidant flavonoids [19]. These compounds play a crucial role in mitigating the detrimental effects of oxidative stress, thereby enhancing the plant’s resilience against pathogen progression. Several flavonoids, including luteolin-7-O-gentiobioside, tricin, flavanone D1, salcolin B, 3-hydroxy-3′-methoxyflavone, galangin, quercetin, naringenin chalcone, and flavanone D2, were significantly elevated in all *MpDIR1(t)* transgenic lines infected with *Pst* DC3000, as detailed in Figure 6A. The high accumulation of flavonoids targets the *Pst* DC3000 type III secretion system and its flagella, significantly decreasing pathogen progression [42].

To further elucidate the variations in the abundance of these flavonoid compounds, a principal component analysis (PCA) was conducted on *MpDIR1(t)* transgenic lines infected with *Pst* DC3000 compared to the WT-I groups. The analysis revealed that the first principal component (PC1) accounted for 73.3% of the total variance observed, while the second principal component (PC2) accounted for 19.7% of the variance, as illustrated in Figure 6B. The PCA plot demonstrated a significant differentiation in the flavonoid profiles, with samples from the WT-I group positioned on the right side of the PC1 axis and those from the *MpDIR1(t)* transgenic lines infected with *Pst* DC3000 aligned on the left side. This distinct separation along the PC2 axis further underscores the differences in flavonoid profiles between the transgenic and wild-type groups (Figure 6B). The PCA clearly explains the distinct clustering patterns observed among the *MpDIR1(t)* transgenic lines and WT-I treatment groups in the leaves of Arabidopsis, emphasizing the prominent variations in the profiles of flavonoid compounds.

### 2.5. Biochemical Attributes of Plants from Transgenic Lines Infected by Pst DC3000

**Figure 7:**
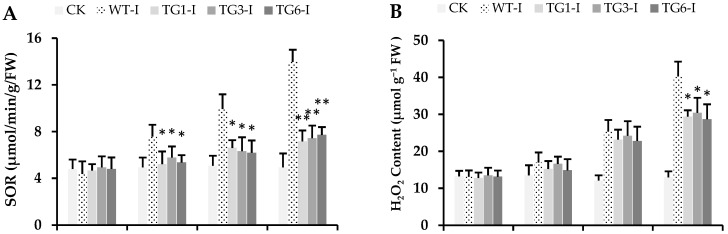

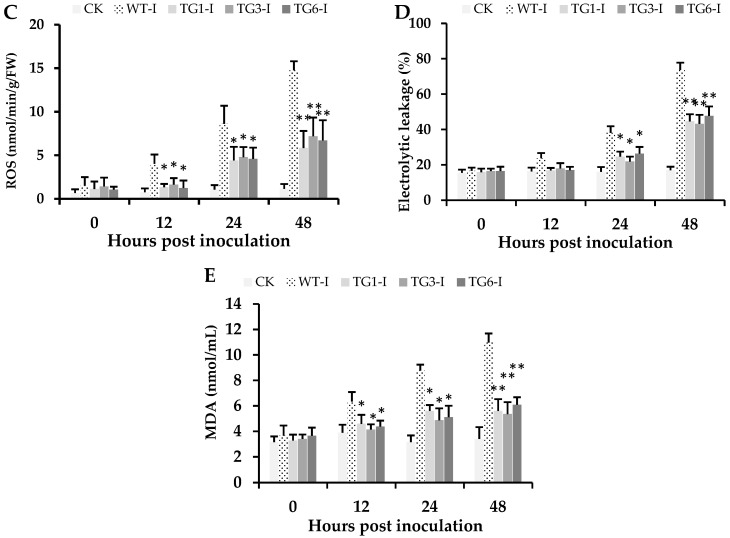
Biochemical variables of *Arabidopsis* leaves infected from *Pst* DC3000. (**A**) Superoxide radicals (SOR), (**B**) hydrogen peroxide (H_2_O_2_) contents, (**C**) reactive oxygen species (ROS), (**D**) electrolytic leakage (%), (**E**) malondialdehyde (MDA). CK: healthy control; WT-I: infected plants from the wild type; TG1-I: infected plants from transgenic line 1; TG3-I: infected plants from transgenic line 3; TG6-I: infected plants from transgenic line 6. Each value is the means of three biological replicates. A Student’s *t*-test was used to compare transgenic *Arabidopsis* expressing *MpDIR1(t)*-TG and WT at * *p* < 0.05 and ** *p* < 0.01.

The contents of superoxide radicals (O_2_^−^), H_2_O_2_, and reactive oxygen species (including H_2_O_2_, O_2_^−^, •OH, and ^1^O_2_) were significantly lower in the transgenic leaves compared to the wild-type leaves following *Pst* DC3000 inoculation (24 to 48 h) (Figure 7A–C). In addition, the wild-type plants exhibited higher levels of electrolytic leakage and malondialdehyde (MDA), indicating that the wild-type leaves experienced more severe stress than the transgenic lines (Figure 7D,E). Generally, plants with lower levels of reactive oxygen species (ROS), MDA, and electrolytic leakage under biotic stress are considered resistant, whereas *Arabidopsis* plants with elevated levels of these markers are deemed as susceptible to *Pst* DC3000 [11,43]. The superoxide radicals, hydrogen peroxide, and malondialdehyde levels significantly increased following both abiotic [44,45] and biotic stresses [43]. Elevated levels of these biochemical markers trigger protein oxidation and cause serve damage to various cellular organelles.

Antioxidant enzymes such as superoxide dismutase (SOD), peroxidase (POD), and catalase (CAT) were significantly induced in transgenic lines challenged by *Pst* DC3000 compared to wild-type *Arabidopsis* (Figure 5B–D). In addition, the antioxidant activity and capacity were significantly increased in the transgenic lines relative to the wild type (Figure 5E,F). Maintaining higher antioxidant levels during progressive stress indicates that these plants possess a durable capability to detoxify the oxidants, such as reactive oxygen species, thereby tolerating stress condition [20,46]. From 0 to 12 h post-inoculation, the antioxidant enzymatic activities increased similarly in both wild-type and transgenic plants (Figure 5B–D). However, from 24 to 48 h post-inoculation, the antioxidant enzymatic activities were significantly higher in all transgenic lines compared to the wild type (Figure 5B–D). Additionally, the wild-type plants exhibited a considerable reduction in enzymatic activities after 48 h post-inoculation; similar trends were observed for antioxidant activity and capacity (Figure 5E,F). Higher antioxidant activity can assist plants in scavenging the ROS generated during biotic stress [43]. Our results indicate that in wild-type plants, the antioxidant defense machinery failed to scavenge ROS effectively after 24 to 48 h of infection by *Pst* DC3000, whereas transgenic plants demonstrated a resilient antioxidant defense mechanism, enabling them to scavenge the oxidants and withstand stress condition.

Previous studies reported that *LTP* genes are not directly involved in SA biosynthesis. However, they are implicated in peptide and lipid signaling, which initiates several defense pathways contributing to high SA accumulation and triggering disease resistance responses in plants [9,47,48]. Earlier research on *Arabidopsis DIR1*-overexpressed transgenic lines indicated the induction of SA-mediated defense signaling [4]. Transgenic *Arabidopsis* lines expressing a *Capsicum annuum LTP* gene demonstrated a positive interaction with SA signaling, triggering the expression of several pathogenesis-related genes, including *PR1*, *PR2*, *PR4*, and *PR5* [34,47]. Our study demonstrates that all transgenic lines exhibit a resilient ability to tolerate *Pst* DC3000 stress by activating several defense genes (Figure 4A–G) compared to wild-type plants. Moreover, all transgenic lines showed higher contents of SA; elevated levels of SOD, POD, and CAT; elevated antioxidant activity and capacity; and lower levels of superoxide radicals, electrolytic leakage, H_2_O_2_, ROS, and MDA after *Pst* DC3000 stress than the wild-type plants. These results revealed that wild-type *Arabidopsis* plants are highly susceptible to *Pst* DC3000 pathogen stress; however, all transgenic lines exhibited resistance to *Pst* DC3000 stress by maintaining lower ROS, MDA, and electrolytic leakage with higher free radical scavenging competence. In this study, we focused on *Arabidopsis thaliana* as a model system, and the effectiveness of *MpDIR1(t)* in citrus species or other crops remains to be tested.

## 3. Materials and Methods

### 3.1. Growing Conditions and Plant Material

Six seedlings of *Murraya paniculata* (Murraya) and *Citrus sinensis* (Sweet orange) at 12 months of age were selected for exposure to citrus greening disease (*Candidatus Liberibacter* asiaticus), whereas three plants from each species were selected as healthy controls. RNA was extracted from *C*Las-positive plants, and the synthesized cDNA was used to perform qPCR. An expression analysis of ten selected genes among six different citrus species (each plant species had six plants) was conducted, and the qPCR primer sequences are provided in Supplementary Table S1. Moreover, the *DIR1* gene was cloned from *Murraya paniculata*, and details of the vector-designing primers are characterized in Supplementary Table S2.

For the development of transgenic lines, *Arabidopsis* wild-type seeds were surface-sterilized with 70% ethanol (*v*/*v*) and grown on Murashige and Skoog (MS) medium (containing 4.43 g of MS-dried basal medium (phyto-technology laboratories), 10 g of agar, and 25 g of sucrose per liter). The seeds were left for three weeks in a growth chamber maintained at 22–24 °C with a 16/8 h light and dark cycle. After one month, *Arabidopsis* plants with flowers were used to overexpress the *MpDIR1(t)* gene via the flower dip method [49].

### 3.2. Agrobacterium Mediated Stable Transformation

The pK7WG2D vector was constructed to overexpress the *MpDIR1(t)* gene in *Arabidopsis* through stable transformation. The pK7WG2D vector contains a green fluorescent protein (GFP) for visual or manual selection of positive plants and confers kanamycin resistance due to the presence of the neomycin phosphotransferase II gene [50]. The *MpDIR1(t)* gene was amplified from cDNA using PCR targeting the coding region. Following plasmid extraction, the gene was cloned into a pDONR221 vector, followed by LR clonase reactions as per the manufacturer’s instructions, and subsequently integrated into the pK7WG2D binary vector for stable transformation using Gateway technology (Invitrogen). The pK7WG2D vector was then transformed into the Agrobacterium strain GV3101 for stable transformation and transferred into *Arabidopsis* via the flower dip method [49] to develop individual transgenic lines expressing the *MpDIR1(t)* gene.

T_0_ *Arabidopsis* seeds were collected and sown on the MS medium containing kanamycin for the manual selection of positive T_1_ stage *Arabidopsis* plants. Confirmation was achieved through DNA extraction and PCR amplification using CaMV35S forward and reverse *MpDIR1(t)* gene-specific primers [51]. Three independent transgenic lines (TGs) of *Arabidopsis* were selected, designated as TG1, TG3, and TG6.

### 3.3. Bacterial Inoculation and Sampling Time

The *Pseudomonas syringae* pv. DC3000 (*Pst* DC 3000) strain was selected to treat both transgenic and wild-type *Arabidopsis* plants. King’s B (KB) medium supplemented with 50 microgram per milliliter of rifampicin antibiotic was utilized to culture the *Pst* DC 3000 bacteria [11]. After 12 h, the log-phase bacterial cultures were optimized to an optimum density among (OD_600_ nm) 0.6–0.8, as measured by a UV-1800 spectrophotometer, where an OD_600_ nm of 0.1 corresponds to 10^8^ colony-forming units (cfu) per milliliter. Prior to infiltration, the bacterial solution was adjusted to a concentration of 10^7^ cfu per milliliter using 10 millimolar MgCl_2_. A 1 milliliter sterile syringe (without needle) was used to infiltrate the bacterial suspensions onto the abaxial surface (inter-veinal space) of *Arabidopsis* leaves. Different time points were selected to evaluate the response of transgenic lines to *Pst* DC3000. For the gene expression analysis, samples were collected at 0, 6, 12, and 24 h post-inoculation, while for the biochemical analysis, samples were collected at 0, 12, 24, and 48 h post-inoculation. At each time interval, the leaves were sampled, immediately frozen in liquid nitrogen, and subsequently stored at −80 °C for further analysis. The *Arabidopsis* plants were cultivated under the following conditions: 70% relative humidity, a temperature of 22 ± 2 °C, a 16/8 h light/dark interval, and a light intensity of 120 micromoles quanta m^−2^ per sec.

### 3.4. DNA Extraction and PCR Analysis

Genomic DNA exaction was performed using the 2% CTAB method [52,53]. Fresh *Arabidopsis* leaves (0.1–0.2 g) were powdered in liquid nitrogen, followed by the addition of 0.7 milliliter of a DNA extraction buffer, and gently inverted for mixing. After incubation at 65 °C for 90 min, 0.8 milliliters of chloroform-isoamyl alcohol (24:1) was added to each DNA sample, mixed thoroughly, and centrifuged for 10 min at 8000 rpm. The supernatant was transferred to a new tube, and an equal volume of isopropyl alcohol was added. Following 30 min of incubation at −20 °C, the DNA pellet was collected by centrifugation at 8000 rpm. The pellet was then washed with 1 milliliter of chilled 100% ethanol and 60 microliter of 5 M NaCl [52]. For amplification of the targeted *DIR1* gene, the PCR-Master mix (Thermo Scientific, Shanghai, China) was used, and the bands were amplified according to the manufacturer’s instructions.

### 3.5. RNA Isolation, cDNA Synthesis, and Quantitative PCR

The TRIzol RNA extraction kit (Takara; Beijing, China) was used to extract RNA from the fresh leaves of *Arabidopsis*. Total RNA was extracted, and complementary DNA (cDNA) was synthesized using the HiScript II QRT reverse transcriptase kit (Vazyme, R223-01; Nanjing, China). Then, 1 microgram (μg) of total RNA was used for cDNA synthesis. The SuperMix (+gDNA wiper) was used to eliminate genomic DNA from each sample, followed by the addition of the reverse transcriptase enzyme according to the manufacturer’s instructions. Post cDNA synthesis, a quantitative-polymerase chain reaction (q-PCR) was conducted using the q-PCR SYBR Green master mix (YEASEN Biotec. Co., Ltd.; Shanghai, China). The β-actin gene from *Arabidopsis* served as an internal reference (control), and all standard procedures were applied as recommended by the producer’s guidelines for q-PCR. The q-PCR analysis was performed using the LightCycler 480 II instrument (Roche Diagnostics, Shanghai, China) to study the expression patterns of different defense genes. Each sample, in triplicate, was loaded onto a 384-well white plate; the LightCycler 480 and the 2−ΔΔCt methodology were employed to calculate the relative expression levels [54]. Detailed information on the q-PCR primers for *Arabidopsis* is provided in the Supplementary Table S3, whereas the primers for *Pst* DC3000 bacterial gene expression [55] are listed in the Supplementary Table S4.

### 3.6. Electrolytic Leakage and Malondialdehyde

The Ohaus Company Starter3100C machine (Shanghai, China) was used to determine electrolytic leakage (EL). Fresh *Arabidopsis* leaves were removed, cut into small sections, and soaked in deionized water. The solution was then left for 20 min at room temperature on a shaker set to 200 rpm. Following the shaking period, EL readings were obtained by immersing the stirrer of the Starter3100C apparatus. Each sample tube was subsequently covered, and the leaf solution was boiled for 15 min. After cooling, additional readings were collected. The EL percentage of each sample solution was calculated using a previously described formula [56].

For the measurement of Malondialdehyde (MDA), the A003-1 kit from Nanjing Jiancheng Bioengineering Institute (Nanjing, China) was utilized. According to the company’s instructions, 0.1 g of *Arabidopsis* leaves was homogenized, and the absorbance of the MDA sample (reaction mixture) was measured using a UV-1800 spectrophotometer (Shimadzu, Kyoto, Japan).

### 3.7. Salicylic Acid Determination

The previous protocol was modified to quantify the salicylic acid (SA) level [57]. Then, 100 milligrams of *Arabidopsis* leaves was homogenized in one milliliter of deionized water. After centrifugation, 500 microliters of the supernatant was transferred to a new tube, and ferric chloride (0.1% freshly prepared) was added to make a total volume of 3 milliliters. The ferric chloride reacted with aqueous SA to form an iron complex, which produces a violet color. This was determined using a UV-1800 spectrophotometer at 540 nm (Shimadzu, Japan). Different concentrations of salicylic acid were used to generate standard curves, whereas deionized water served as the blank control.

### 3.8. Hydrogen Peroxide (H_2_O_2_) Determination

H_2_O_2_ determination was performed according to previous protocol [58]. Briefly, 0.1 g of *Arabidopsis* leaves were grounded into powder, followed by the addition of 1 mL of 1% trichloro-acetic acid, and mixed well in an ice bath for 10 min. After incubation in the ice bath, the samples were centrifuged at 10,000 rpm for 8 min at room temperature, and the supernatant was collected into fresh tube. The reaction mixture contained 0.5 mL of the supernatant mixed with an equal volume of potassium phosphate buffer (10 mM concentration). Finally, 1 mL of 1 M potassium iodide was added, followed by vortexing. For the standard curve, commercial H_2_O_2_ was used. The absorbance of each sample and standard reaction mixture was measured using a UV-1800 spectrophotometer (Shimadzu, Japan) at 390 nm, and the H_2_O_2_ concentration was determined as micromoles/gram of each sample.

### 3.9. Superoxide Radicals and Reactive Oxygen Species Assay

From each sample, 0.1 g of *Arabidopsis* leaves was homogenized to measure superoxide radicals (O_2_^−^) [59]. A 0.1 unit change in absorbance was considered as one unit of O_2_^−^/min at the corresponding wavelength, as previously described [59]. For the determination of reactive oxygen species, including H_2_O_2_, O^2−^, hydroxyl radicals (•OH), and singlet oxygen (^1^O_2_), a kit (Elabscience: CAT No: E-BC-K138-F; Wuhan, China) was used according to the manufacturer’s guidelines.

### 3.10. Antioxidant Activity and Capacity (DPPH Free Radical Scavenging Assay)

To calculate antioxidant capacity and activity, the 2,2-diphenyl-1-picrylhydrazyl (DPPH 0.1 mM) method was used [60]. *Arabidopsis* leaves (0.1 g) were homogenized in 1 mL of reaction solution having 70% ethanol, 29% water, and 1% acetic acid, followed by centrifugation for 8 min at 8000 rpm. The supernatant (0.03 mL) was collected into a new tube, and 2.97 mL of DPPH solution was added to each sample. The mixture was then incubated in the dark at room temperature for 30 min. A reaction mixture without the sample served as the control. Absorbance of each sample was measured using a UV-1800 spectrophotometer (Shimadzu, Japan) at 517 nm. Trolox was used to generate the standard curve, and the antioxidant capacity was expressed in millimolars of trolox equivalents/100 mg. Antioxidant activity (free radical scavenging percentage) was calculated using the following formula:

Antioxidant activity (%) = [1 − {sample OD/control OD}] × 100.

### 3.11. Antioxidant Enzymatic Activity

The antioxidant enzymatic activities, including superoxide dismutase (SOD), peroxidase (POD), and catalase (CAT), were observed in the *Arabidopsis* leaves using kits purchased from Nanjing Jiancheng Bioengineering Institute (Nanjing, China). The following kits were employed for the respective enzymatic assays: SOD (A001-1-1), POD (A084-3-1), CAT (A007-1-1), and total protein content (A045-2-2). Protein contents and enzymatic activities were measured using 500 mg of *Arabidopsis* leaves, following the manufacturer’s protocol. In addition, control, standard, and sample readings were taken using a UV-1800 spectrophotometer.

### 3.12. Flavonoid Profiling in Transgenic Arabidopsis Leaves

Flavonoid compounds in transgenic *Arabidopsis* leaf samples were examined using ultra-performance liquid chromatography–tandem mass spectrometry (UPLC-MS/MS). Freeze-dried transgenic *Arabidopsis* leaf samples were pulverized using liquid nitrogen, with 50 mg of the resulting powder used for both qualitative and quantitative analysis. To each powdered sample, 500 μL of 70% aqueous methanol was added, followed by ultrasonic extraction (Model: KQ5200E) at 5 °C for 30 min. Centrifugation was then conducted at 12,000 rpm for 5 min. The resulting supernatant was collected and passed through a 0.22 μm membrane into a fresh centrifuge tube. This filtrate was then analyzed using UPLC-MS/MS and multiple reaction monitoring, operating in both negative 4500 and positive 5500 ion modes, as previously described [20,61,62]. Rutin, at a volume of ten microliters, served as the internal standard. Analyst 1.6.3 software was used for analysis of the mass spectrum data.

### 3.13. Statistical Analysis

All data were statistically analyzed using Statistix 8.1 statistical software (Tallahassee, FL, USA; Analytical Software). The Excel program (Microsoft Corp., Redmond, WA, USA) was used for calculating standard errors and developing graphs. Hierarchical cluster analysis (HCA) and principal component analysis (PCA) were performed on the *MpDIR1(t)* transgenic lines and WT, both infected with *Pst* DC3000. The data were min–max normalized, as described before [63]. GeneDoc (version 2.6) software was used for the amino acid sequence analysis [64], whereas MEGA7 and TBtools were used for generating phylogenetic trees [65,66].

## 4. Conclusions

Our study concluded that the *DIR1* gene was significantly induced, more than 12-fold, in *C*Las-resistant *Murraya paniculata* after *C*Las inoculation compared to the *C*Las-susceptible *Citrus sinensis DIR1* gene, which showed less than a 2-fold induction. Moreover, overexpression of the *MpDIR1(t)* gene significantly induced defense-responsive genes and salicylic acid biosynthetic genes in all transgenic *Arabidopsis* lines. In addition, all transgenic *Arabidopsis* lines exhibited resistance to *Pst* DC3000. Our gene expression results revealed that many genes associated with defense, such as *PR1*, *PR2*, *PR5*, *PR10*, *WRKY12*, and *PAL* were significantly up-regulated 2–12 folds in transgenic *Arabidopsis* lines infected with *Pst* DC3000 compared to the wild type. Furthermore, the transgenic *Arabidopsis* lines showed lower levels of reactive oxygen species and superoxide radicals, as well as higher levels of antioxidant flavonoids and enzymatic activities compared to the wild type. The gene expression, phenotypic, biochemical, SA, and antioxidant enzymatic results determined that the *MpDIR1(t)* gene conferred resistance to *Pst* DC3000. The *Murraya paniculata DIR1* gene is a promising candidate for increasing bacterial diseases resistance in susceptible citrus cultivars and contributing to the healthy development of the citrus industry.

## Data Availability

All data are available in the manuscript and Supplementary Materials.

## Supplementary Materials

The following supporting information can be downloaded at https://www.mdpi.com/article/10.3390/plants13192692/s1, Figure S1: The gene expression pattern of the *MpDIR1* gene in overexpressed Arabidopsis lines compared with the wild type. CK: healthy control; WT: wild type; TG1: transgenic line 1; TG3: transgenic line 3; TG6: transgenic line 6. Each value is the mean of three biological replicates. A Student’s *t*-test was used to compare transgenic Arabidopsis lines expressing *MpDIR1*-TG and WT at ** *p* < 0.01. Figure S2: The gene expression pattern of different genes associated with the pathogen-responsive and SA-mediated defense pathways. (A) *AtPI2*, (B) WRKY1, (C) CBP60G, (D) *AtNPR1*. At: *Arabidopsis thaliana*; CK: healthy control; WT-I: infected plants from the wild type; TG1-I: infected plants from transgenic line 1; TG3-I: infected plants from transgenic line 3; TG6-I: infected plants from transgenic line 6. Each value is the mean of three biological replicates. A Student’s *t*-test was used to compare transgenic Arabidopsis expressing *MpDIR1*-TG and WT at * *p* < 0.05 and ** *p* < 0.01. Table S1: The primer sequences used for the gene expression analysis in six different species of citrus. Table S2: The cloning and *MpDIR1(t)*-Vector construction primers used to amplify the *DIR1* gene from Murraya. Table S3: The primer sequences used for gene expression analysis of Arabidopsis. Table S4: The primer sequences used to monitor the *Pst DC3000* gene expression analysis.
