# Supplementary material for: The Ectopic Expression of the MpDIR1(t) Gene Enhances the Response of Plants from Arabidopsis thaliana to Biotic Stress by Regulating the Defense Genes and Antioxidant Flavonoids
Source: Plants (Basel). 2024 Sep 25;13(19):2692. doi: 10.3390/plants13192692 (PMC11478391; doi:10.3390/plants13192692)
Supplement: Supplementary file 1 [file plants-13-02692-s001.zip › plants-3141037-supplementary.pdf]

Table S1: Representing the primer sequences used for gene expression analysis in six different species of citrus.

Serial No	Primer Name/ IDs	Primer Sequence (5'to3')	Primer length
1	DIR1f	AGGCTCTTGCTGGTGCTGA	19
2	DRI1r	ACATTGGGGACCCTTTGTAAC	21
3	ATf	TCAGTGCGATTTTGGGACC	10
4	ATr	AGCCACCGACCGATGCTGAT	20
5	NAC096f	GCTTATCTCCTTGATTGAGTTCG	23
6	NAC096r	GCAAGAGCAACCAGAACTGAC	21
7	WRKY40f	CTTGGCTTTGTGGGTTTGC	19
8	WRKY40r	TGCTCCCCTTCGTAAGTCGC	20
9	GPT2f	TAGCCCATACTTTACGTCATCTC	23
10	GPT2r	AACCAGCAACACCATCACTAA	21
11	F3-Of	TCAGCCCCGACCTCACATA	19
12	F3-Or	GGCGTTTGATGATTGAAAGAAC	22
13	WRKY53f	AGACTTTGCCAAGGAGGACG	20
14	WRKY53r	TTTCTGTAGGTGCATCTGTAATAGC	25
15	MKK9f	CACGGAAACGGTGGAACAG	19
16	MKK9r	AGTGCGGCGGAGGATTT	17
17	WRKY33f	TCATCCCAAGCCTCAATCTACA	22
18	WRKY33r	GGATGGCATTAGAATTAACCGA	22
19	WRKY70f	CCCTTTCATTATCACCATCG	20
20	WRKY70r	CAGAAGCCACCTATGACCCT	20

Table S2: Representing the Cloning and DIR1-Vector construction primers to amplify the *DIR1* gene from Murraya.

Serial No	Primer Name	Primer Sequence (5'to3')	Primer length	Primer type	Product size
1	Forward DIR1	ATGGAGATGGGAAAGAAGTT	20	Cloning	300bp
2	Reverse DRI1	TTAGGAACATGGAACAGCAA	20		
3	attB1+DIR1-F	AAAAAGCAGGCTTCATGGAGATGGGAAAGAAGTT	34	Vector Preparation	320bp
4	attB2+DIR-R	AGAAAGCTGGGTGTTAGGAACATGGAACAGCAA	33		

Table S3: Showing the primers sequences used for gene expression analysis of Arabidopsis.

Serial No	Primer Name	Primer Sequence (5'to3')	Arabidopsis gene IDs
1	PR1f	5' AAACTTAGCCTGGGGTAGCG 3'	AT2G14610
2	PR1r	5' CACCTCACTTTGGCACATCC 3'	
3	PR2f	5' CTACGGGATGCTAGGCGATAC 3'	AT3G57260
4	PR2r	5' CGTTTGACTGGAGGCGAGA 3'	
5	PR4f	5' ATAGACACCGATGGTTTTGGC 3'	AT3G04720
6	PR4r	5' GCATGTTTCTGGAATCAGGCT 3'	
7	PR5f	5' ATTGACTCCAGGTGCTTCCC 3'	AT1G75040
8	PR5r	5' CGCCGCCGTTACATCTTAG 3'	
9	PR10f	5' GGTCAGATCAGCAGCGACAA 3'	AT3G04710
10	PR10r	5' CATCCGTTAGATTCGCCTGTA 3'	
11	AtNPR1f	5' CTACCGATAACACCGACTCCTC 3'	AT1G64280
12	AtNPR1r	5' TCGCTGACAAAACGCACC 3'	
13	AtWRKY1f	5' TGAGAAGGTTATGGAAGACGGAT 3'	AT2G04880
14	AtWRKY1r	5' CAGCAAGAGGCTTTGGGTG 3'	
15	AtWRKY12f	5' TGACGATGGCTACAAATGGC 3'	AT2G44745
16	AtWRKY12r	5' TCCTGGGGTGAAGGCTGTT 3'	
17	AtPALf	5' TTGGATTATGGATTCAAGGGAG 3'	AT2G37040
18	AtPALr	5' TCAGAAGTTTTGCGAGACGAG 3'	
19	AtPI1f	5' GGGACTGAAGGCTTTGGAAC 3'	--
20	AtPI1r	5' TGATGACCTGGCAACTTCTCC 3'	
21	AtPI2f	5' AACGACGGAAACATCGGACT 3'	--
22	AtPI2r	5' TCTTCACCACCAGGAGCAAAC 3'	
23	CBP60G9	5' GTTCTCGTCTTCTCGGGTCG 3'	AT5G26920
24	CBP60G9	5' TCACCGTTAGGTCTCCAGTGAG 3'	
25	Atactinf	5' CCCAGGTTGAGAAAGAAATCG 3'	--
26	Atactinr	5' CTCGCAGTCTGCTGTGACGT 3'	

*At *Arabidopsis thaliana*

Table S4: Showing the primers sequences used to monitor *Pseudomonas syringae DC3000* gene expression analysis.

ID	Primer	Primer Sequence (5'to3')	Product size	Primer length
1	QRT-ps16sV1-F	ACGGGTACTTGTACCTGGTG	87bp	20
2	QRT-ps16sV2-R	CGTTTCCGAGCGTTATCCC		19

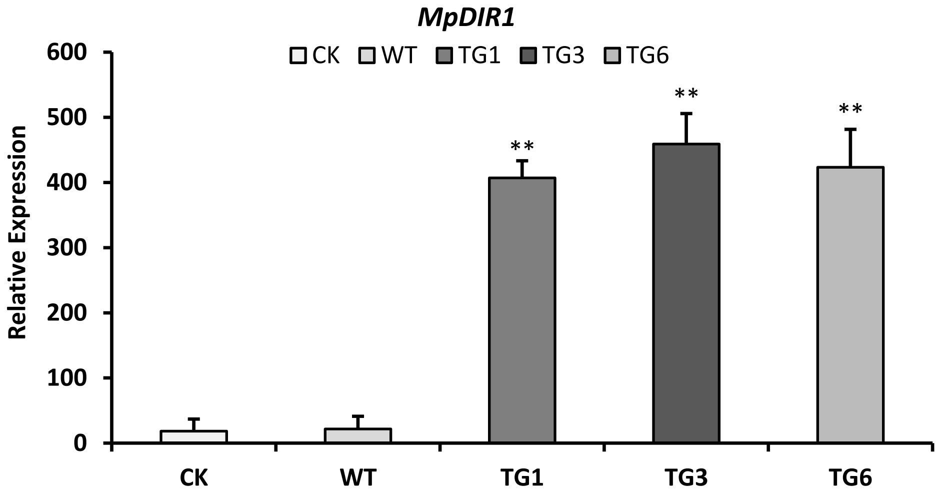

Figure S1: Gene expression pattern *MpDIR1* gene in overexpressed Arabidopsis lines compared with wild type. CK: Healthy control, WT: Plants from wild-type, TG1: Plants from transgenic line 1, TG3: Plants from transgenic line 3, TG6: Plants from transgenic line 6. Each value is mean of three biological replicates. Students t-test was used to compare transgenic Arabidopsis lines expressing *MpDIR1*-TG and WT at $^{**}p < 0.01$.

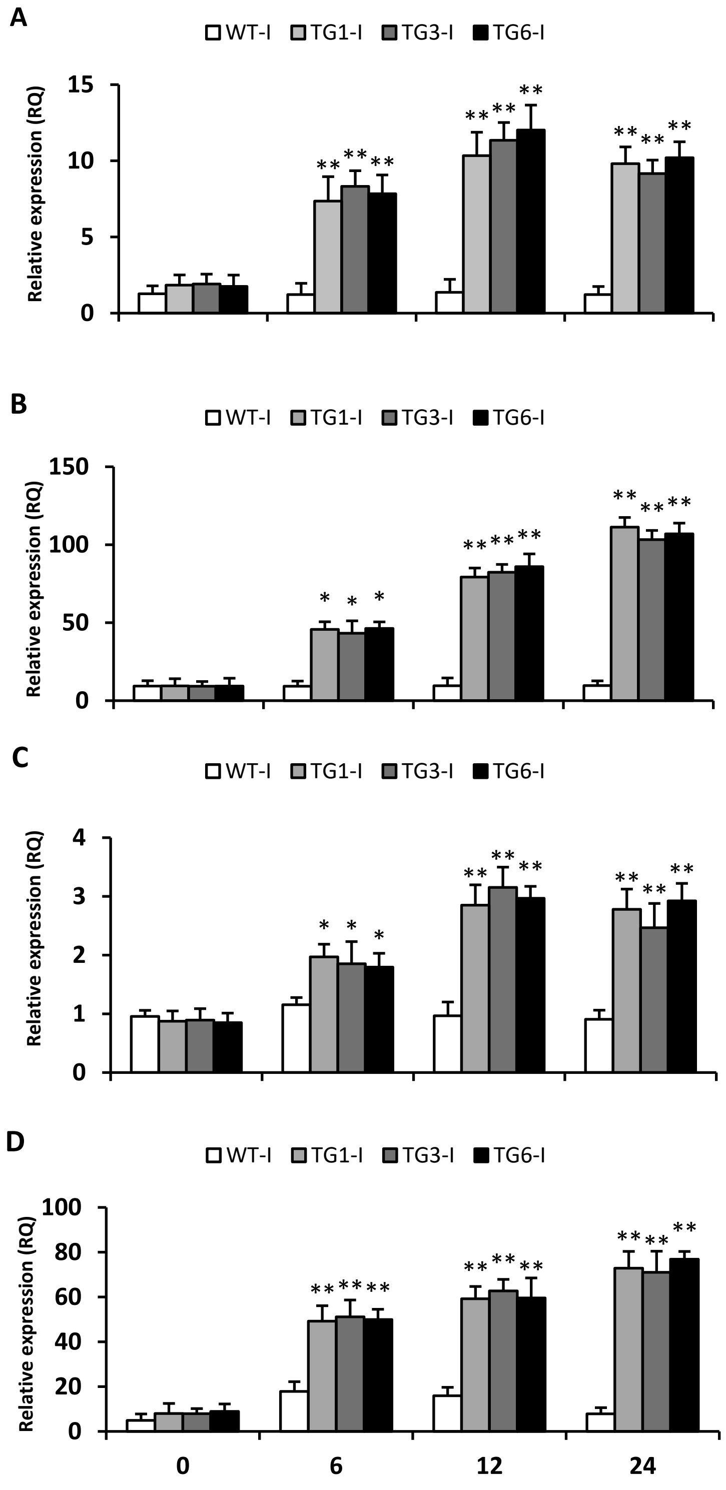

Figure S2: Gene expression pattern of different gene associated with pathogen responsive and SA-mediated defense pathway. (A) AtPI2 (B) WRKY1 (C) CBP60G (D) AtNPR1. At: *Arabidopsis thaliana*, CK: Healthy control, WT-I: Infected plants of wild-

type, TG1-I: Infected plants of transgenic line 1, TG3-I: Infected plants of transgenic line 3, TG6-I: Infected plants of transgenic line 6. Each value is mean of three biological replicates. Students t-test was used to compare transgenic Arabidopsis expressing *MpDIR1*-TG and WT at *$p < 0.05$; **$p < 0.01$.
